# Identification, cloning and characterization of an ultrapetala transcription factor *CsULT1* from *Crocus*: a novel regulator of apocarotenoid biosynthesis

**DOI:** 10.1186/s12870-015-0423-7

**Published:** 2015-02-01

**Authors:** Nasheeman Ashraf, Deepti Jain, Ram A Vishwakarma

**Affiliations:** Plant Biotechnology Division, CSIR- Indian Institute of Integrative Medicine, Sanat Nagar, Srinagar, J&K-190005 India; National Institute of Plant Genome Research, Aruna Asaf Ali Marg, New Delhi, 110067 India; Medicinal Chemisrty Division, CSIR- Indian Institute of Integrative Medicine, Canal Road, Jammu, J&K 180001 India

**Keywords:** Ultrapetala, Crocus, Stigma, Saffron, Carotenoids, Apocarotenoids, SAND domain

## Abstract

**Background:**

*Crocus sativus* is a triploid sterile plant with long red stigmas which form commercial saffron. Saffron is the site for synthesis and accumulation of apocarotenoids like crocin, picrocrin and safranal which are responsible for its color, flavour and aroma making it world’s most expensive spice. These compounds are formed by oxidative cleavage of zeaxanthin by carotenoid cleavage dioxygenases. Although the biosynthetic pathway of apocarotenoids is known to a considerable extent, the mechanism that regulates its tissue and developmental stage specific expression is not known.

**Results:**

In the present work, we identified, cloned and characterized ultrapetala transcription factor called *CsULT1* from *Crocus*. The gene contains an 80 amino acid long conserved SAND domain. The *CsULT1* transcript was more abundant in stigma and showed increase in expression from pre anthesis stage till anthesis and decreased in post anthesis stage which corroborated with the accumulation pattern of crocin indicating its possible role in regulation of apocarotenoid biosynthesis. *CsULT1* was found to be transcriptionally active and localized in nucleus. Its expression is induced in response to phytohormones like auxin, methyljasmonate and salicylic acid. Overexpression of *CsULT1* in *Crocus* calli resulted in enhanced expression of key pathway genes like *phytoene synthase* (*PSY*), *phytoene desaturase* (*PDS*), *beta carotene hydroxylase* (*BCH*) and *carotenoid cleavage dioxygenases* (*CCDs*) indicating its role in regulation of apocarotenoid biosynthesis.

**Conclusion:**

This work presents first report on isolation and characterization of ultrapetala gene from *Crocus.* Our results suggest that *CsULT1* is a novel regulator of *Crocus* apocarotenoid biosynthesis. We show for the first time involvement of plant SAND domain proteins in regulating secondary metabolic pathways.

**Electronic supplementary material:**

The online version of this article (doi:10.1186/s12870-015-0423-7) contains supplementary material, which is available to authorized users.

## Background

*Crocus sativus* L. (Iridaceae) is a sterile triploid plant propagated vegetatively through corms [1]. The desiccated stigma of *C. sativus* forms saffron and is source of various carotenoids and unique compounds called apocarotenoids which are produced by oxidative tailoring of carotenoids [2]. Apocarotenoids are synthesized in a number of plants including maize, tomato, *Arabidopsis*, *Crocus* etc. but *Crocus* finds a special place because it is the only plant which produces crocin, picrocrocin and saffranal in significant quantities [3]. The saffron apocarotenoids are formed by zeaxanthin cleavage [4] followed by specific glycosylation steps [5]. Because of the presence of these unique apocarotenoids *Crocus* stands apart from other related crops and is considered as one of the world’s costliest spices [6]. Besides, saffron apocarotenoids also have tremendous pharmacological properties and have been used for the treatment of a wide range of cancers [7,8].

Carotenoids and their cleavage products are synthesized by plastid localized methylerythritol phosphate (MEP) pathway. Biosynthesis of these compounds is regulated throughout the life cycle of a plant and dynamic changes occur in their composition to match the prevailing developmental requirements and response to external environmental stimuli [9]. Although the carotenoid biosynthetic pathway has been studied to a considerable extent in many plants including *Crocus* but the fundamental knowledge regarding the regulation of carotenogenesis in plant cells is still in its infancy [10]. In *Crocus*, apocarotenoids are synthesized only in stigma part of the flower and that too in developmental stage specific manner, but nothing is known about the mechanism that regulates its synthesis. Therefore, it will be quite interesting to take a step towards unravelling the regulatory pathway of carotenoid/apocarotenoid biosynthesis in *Crocus*.

The major goal of the present study was to identify transcription factors that regulate apocarotenoid biosynthesis in saffron. It is a well established fact that stigma part of the *Crocus* flower is the actual site for synthesis of many important apocarotenoids [11,12], however, we still attempted to study pattern of crocin accumulation (crocin being an important metabolite) in different parts of the flower and at different stages of stigma development. We also selected five transcription factors belonging to five different gene families from saffron gene database [1] and investigated their temporal and spatial expression profile. The results demonstrated that *ULTRAPETALA* (*ULT*) gene shows higher expression in stigma tissue and the expression increased till the day of anthesis and subsequently decreased. This expression profile matched with the accumulation pattern of crocin in saffron thereby indicating a possible role of this gene in regulating biosynthesis of apocarotenoids. The *ULT* encodes a small cysteïne rich protein containing a B-box like motif and a SAND domain, a DNA binding motif previously reported only in animal transcription factors [13]. This transcription factor has been proposed to act as regulator of developmental gene expression. In *Arabidopsis*, it functions in floral stem cell termination pathway [14]. *ULT* has been shown to act as trithorax group chromatin remodelling factor which regulates function of Agamous locus during stem cell termination [15]. Recently it has been demonstrated that *ULT1* acts as an antirepressor that promotes transcriptional activation by antagonizing PcG-induced histone methylation and, via physical interaction with *ATX1* that deposits H3K4me3 activating marks, promotes an open chromatin conformation to recruit proteins involved in transcriptional initiation and elongation [16]. More recently *ULT* was found to be involved in gynoecium formation [17]. *ULT1* function thus represents a novel chromatin-mediated mechanism that activates genes controlling stem cell fate in plants. This observation expands the repertoire of plant epigenetic regulators involved in developmental pathways and suggests involvement of chromatin mediated pathways in controlling dynamics of transcription during such pathways.

In this report we describe identification, isolation and characterization of *ULT* gene, *CsULT1*, from *Crocus sativus. CsULT1* is preferentially expressed in stigma and induced by phytohormones such as MJ, SA, 2,4-D. Further, *CsULT1* is localized in nucleus and is transcriptionally active. *Crocus* transformation has not yet been established. Here we studied transient overexpression of *CsULT1* in *Crocus* calli and observed that its overexpression upregulates some key carotenoid/apocarotenoid pathway genes. This work represents, to our knowledge, the first functional characterization of a *C. sativus ULT* gene and also first report on a transcriptional regulator of apocarotenoid biosynthetic pathway.

## Methods

### Plant material

*Crocus sativus* was grown in the experimental farm at Indian Institute of Integrative medicine (IIIM), Srinagar, India (longitude: 34°5′24′′N; latitude: 74°47′24′′ and altitude 1585 m above sea level). It was used as source plant material for the present study. The voucher specimen was deposited at Janaki Ammal Herbarium (RRLH), IIIM, Jammu. The details of the specimen are: (Accession number: 22893; Accession date: 12/01/2015; name of collector: Nasheeman Ashraf; Place of collection: IIIM, Srinagar Farm; Date of collection: 01/01/2015). For tissue specific expression profiling, on the day of flower opening, tepals, anthers and stigma were collected from flowers separately, frozen in liquid nitrogen and stored in −80ºC till further use. For developmental stage specific expression, stigma was collected at three different stages viz three days before anthesis, on the day of anthesis and two day after anthesis. For hormone treatments, flowers were grown in pots and were mist sprayed with 100 μM methyljasmonate, 1 mM salicylic acid, 50 μM 2, 4-D and 100 μM ABA. Tissue samples were collected after 12 and 24 h of hormone treatment. For overexpression studies, calli overexpressing *CsULT1* and vector control calli were taken for RNA isolation. For each experiment, tissue from three biological replicates was pooled in.

### Sample preparation and HPLC analysis

Crocin analysis was done as described by Moraga *et al.* [12]. For extract preparation, 0.5 mg tissue from tepals, anthers and stigma (collected at three different stages) was crushed with a micropestle in 700 μl Tris–HCl (50 mM, pH 7.5 containing 1 M NaCl), and incubated for 10 minutes on ice. This was followed by addition of 700 μl of chloroform. The extract was then incubated on ice for an additional 10 min. Centrifugation at 3000 g for 5 min at 4°C was done to separate the phases. The lower chloroform phase was evaporated and the dried residues were stored together with the upper aqueous phases at −80°C until high-performance liquid chromatography (HPLC) analysis. The LCMS apparatus of Nexera UHPLC (130 MPa) equipped with MS-8030 (Shimadzu) was used for the Study and data was generated using lab solutions software. Enable RP-C18 column (250 mm × 4.6 mm, 5 μm) was used. The injection volume was 5 μl and flow rate 0.3 ml/min. Mobile Phase A (Water and Acetonitrile ratio 1:1) and mobile phase B (0.1% Acetic acid in water) were used in a linear gradient flow and column temperature was set at 75°C initially.

### Gene expression analysis using quantitative real time PCR

Total RNA was extracted from pooled tissue using TRIzol reagent and used for cDNA synthesis by Reverse Transcription kit (Fermentas) following manufacturer’s instructions. qRT-PCR was performed in triplicates in ABI StepOne Real time (Applied biosystems). The reaction was carried out in a total volume of 20 μl, consisting of 10 μL of 2X SYBR Green Master Mix, 0.2 μM (each) gene specific primers for all the genes studied and 100 ng of template cDNA. The cycling parameters were 95°C for 20 s, followed by 40 cycles of 95°C for 15 s and 60°C for 1 min. The sequence of all the primers used in this study is given in Additional file 1. The specificity of each primer pair was validated by a dissociation curve (a single peak was observed for each primer pair) (Additional file 2). The relative quantification method (ΔΔ-CT) was used to evaluate quantitative variation between the replicates examined. The amplification of actin cDNA was used as an endogenous control to normalize all data.

### Cloning of full length *CsULT1* gene

The partial clone of *CsULT1* was obtained using cDNA synthesized from *Crocus* flower RNA and primers (ULT-F and ULT-R) designed from EST sequence (cr.saCl000502:1) present in NCBI (http://www.ncbi.nlm.nih.gov/nucest). Sequence analysis of the partial clone revealed that it has the 3’end and only 5’end needs to be amplified in order to obtain the full length clone. Thus the full length cDNA clone of *CsULT1* was obtained by performing 5′RACE using gene specific primer (ULT-5’) and UAP primer provided with the 5′RACE kit (Clontech) following manufacturer’s instructions. The amplified product was run on 1% agarose gel and purified with gel extraction kit (Qiagen). The purified product was then cloned in the pGEM-T Easy vector and sequenced. For the amplification of full length clone, gene specific primers were designed from the full length nucleotide sequence as obtained from alignment of partial clone and the 5’RACE product. The full length cDNA clone was amplified by PCR using cDNA as template and the gene specific primer pair (CsULT-F and CsULT-R). The PCR product was run on 1% agarose gel, purified by gel extraction kit (Qiagen) and subsequently cloned into the pGEM-T Easy vector. The cycling conditions used were 3 min at 94°C, 30 cycles (30s at 94°C, 30 s at 60°C and 1 min at 72°C) and final extension for 10 min at 72°C. The nucleotide sequence of *CsULT1* was submitted to GenBank and the accession number is KM670459.

### Sequence analyses

The full length nucleotide sequence of *CsULT1* was translated using Translate tool (http://web.expasy.org/translate/) and the properties of deduced amino acid sequence were estimated using ProtParam (http://web.expasy.org/protparam/). Multiple sequence alignment and phylogenetic analysis was performed using the ClustalW with the default parameters through the service of the European Bioinformatics Institute (http://www.ebi.ac.uk/Tools/msa/clustalw2).

### Subcellular localization

The subcellular localization of *CsULT1* was studied by performing transient expression assay in onion epidermal cells. For this, *CsULT1* with restriction sites for *Nco*I and *Spe*I was amplified using ULTCam-F and ULTCam-R1 primer pair and fused in frame with 5’ terminus of GFP reporter gene in pCAMBIA-1302. The cycling parameters were same as described above. The fusion construct of *CsULT1*-GFP was bombarded on to the onion peels using biolistic gene delivery device PDS-1000/He (Bio-Rad, USA). The onion peels were then incubated for 24 hours before visualizing in confocal microscope.

### Transactivation assay

Full length protein coding sequence of *CsULT1* was cloned in yeast (*Saccharomyces cerevisiae*) expression vector pGBKT7 (Clontech) at *NdeI-EcoRI* site to express CsULT1 protein fused to GAL4 DNA-binding domain (GAL4-BD). The primers used were ULTGBKT-F and ULTGBKT-R and cycling parameters are same as described above. The resulting construct was transformed into Y187 yeast strain. The positive transformants were selected onto synthetic medium lacking tryptophan and leucine. Cells from two independent transformants were collected and assayed for β-galactosidase activity by using ortho-nitrophenyl-β-D-galactoside (ONPG) as substrate as described in clontech manual (PT3024-1).

### Plant expression vector and transformation of *Crocus* calli

The *CsULT1* gene with *NcoI* and *SpeI* restriction sites was PCR amplified using ULTCam-F and ULTCam-R2 primer pair and cloned into pCAMBIA1302 vector, containing CaMV 35S promoter. For the transient expression, the *Crocu*s calli were arranged at the center of petri dish and the biolistic gene delivery device PDS-1000/He (Bio-Rad, USA) was used for transgene delivery via microprojectile bombardment. Plasmid DNA (at the concentration of 1 μg/μL) was coated on the surface of gold particles and bombarded on to the calli. The particle delivery system was adjusted to 1100 psi of helium pressure and 27 mmHg of vacuum pressure inside the chamber. After bombardment, the calli were transferred to fresh media and after five days they were again transferred to media containing hygromycin for selection of transgenic structures. After 10 days, the calli which did not harbour the *CsULT1*-pCAMBIA construct turned blackish whereas the ones with the gene construct looked fresh and were used for further experimental studies. The transgenic calli were screened by genomic PCR. For this, genomic DNA was isolated from independent *CsULT1* overexpression and empty control calli using the DNeasy Plant mini kit (Qiagen). The presence of *CsULT1* was confirmed by genomic PCR using gene specific primer and reverse primer corresponding to GFP. Transgenic and control calli were used for measuring the transcript levels of few carotenoid pathway genes including *PSY* (GenBank accession: AJ888514), *PDS* (GenBank accession: AY183118)*, BCH* (GenBank accession: AJ937791). *CCD4b* (GenBank accession: EU523663.1) and *CCD2* (GenBank accession: KJ541749) using quantitative real time PCR as described above.

## Results

### Analysis of Crocin in different tissues and developmental stages

**Figure 1:**
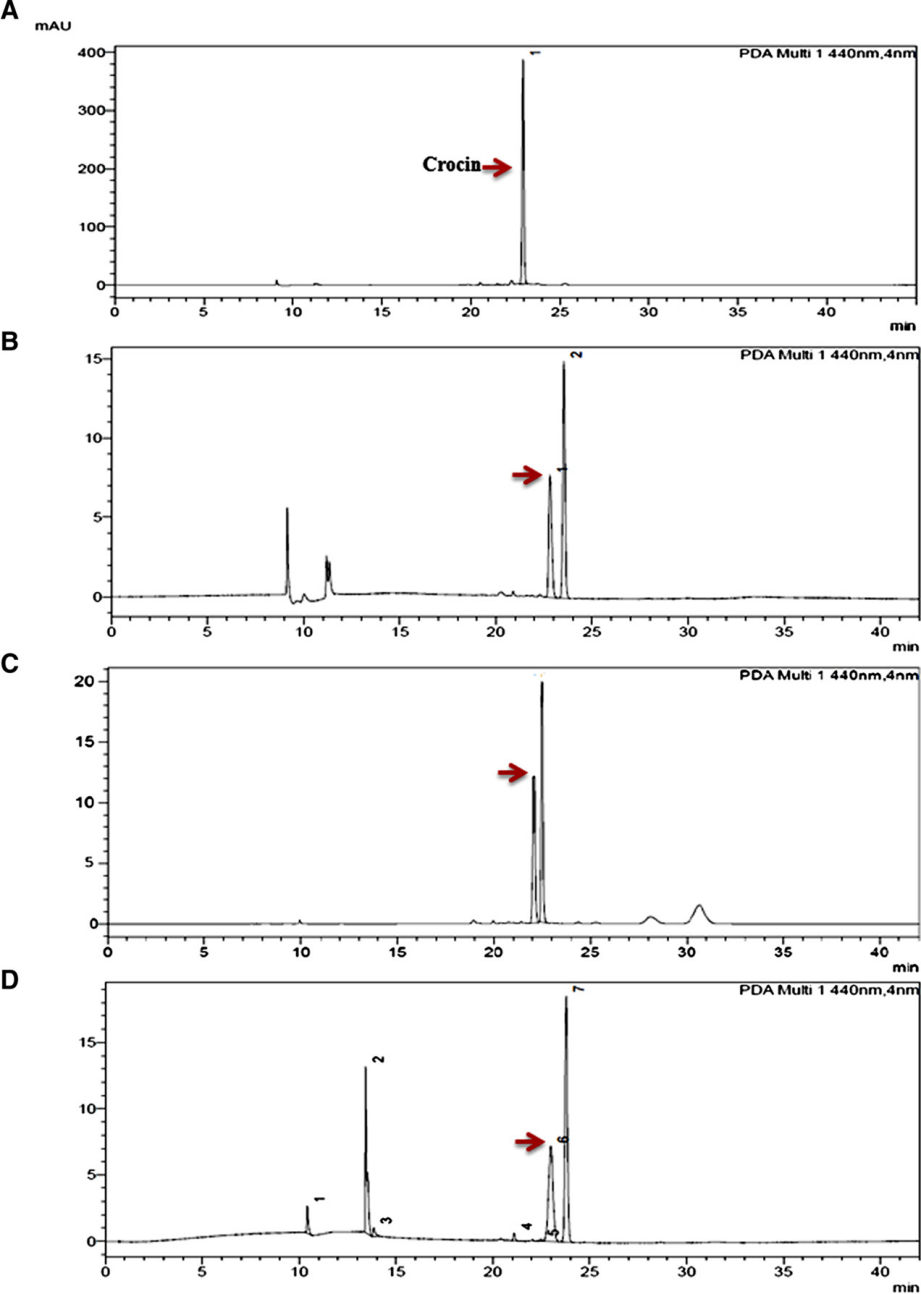
**HPLC chromatograms for crocin. (A)** represents chromatogram for crocin standard, **(B-D)** chromatogramas for *Crocus* stigma collected at pre anthesis, anthesis and post anthesis stages.

Since crocin is the most important metabolite in saffron and responsible for its coloring property, we measured its quantity in different parts of *Crocus* flower and at various stages of stigma development (pre anthesis, anthesis and post anthesis) using HPLC. Results indicated that crocin was present only in stigma part of flower. We were not able to detect crocin in other parts of the flower like tepals and anther. Further, its content showed increasing trend from pre anthesis to anthesis stage and later again decreased after anthesis (Figure 1). This was in confirmation with earlier reports [12] where they have shown that the major apocarotenoids like crocin and picrocrocin are detected in orange stage and increased rapidly during the following stages of stigma development till they reached maximum in scarlet stage at anthesis. This confirms that stigma is the site for synthesis and accumulation of major *Crocus* apocarotenoids and their synthesis is congruent with development of stigma reaching highest at anthesis stage.

### Isolation and expression profiling of *CsULT1*

**Figure 2:**
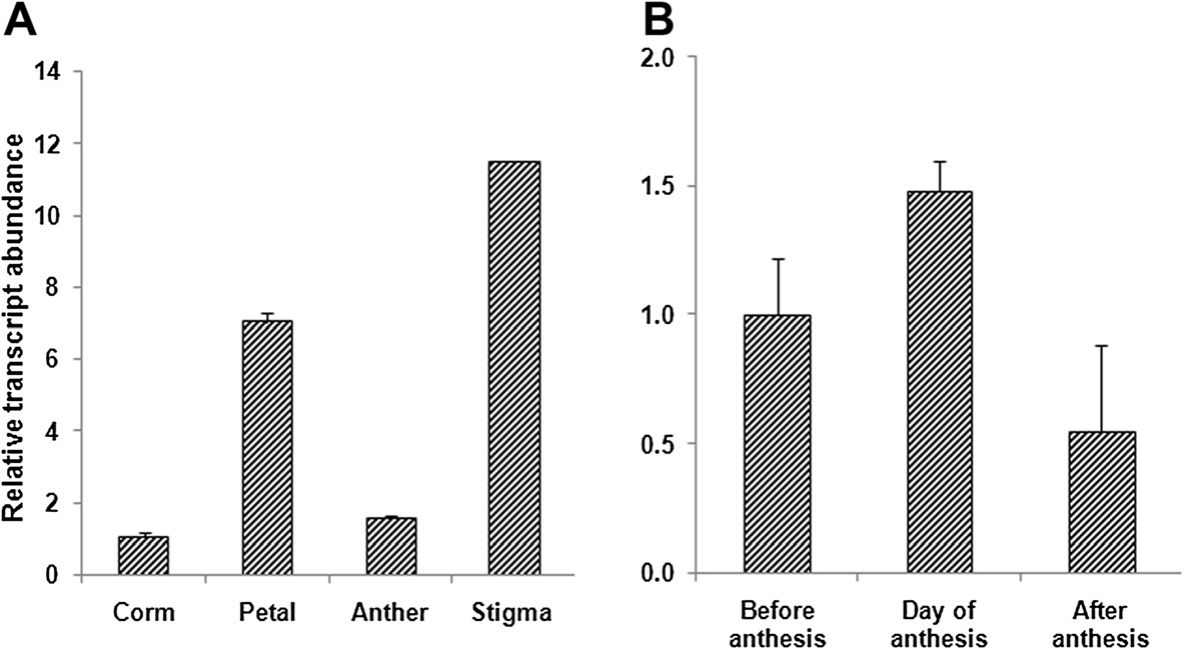
**qRT-PCR analysis of** ***CsULT1*** **expression. (A)** in different tissues of *C. sativus* **(B)** at different developmental stages. Transcript levels were normalized by actin. Data are means and SD from three biological replicates.

We aimed at identification of transcription factors which regulate biosynthesis of *Crocus* apocarotenoids. Towards this, five transcription factors belonging to different gene families were selected from saffron ESTs (http://www.ncbi.nlm.nih.gov/nucest). The selected genes were Myb (cr.saCl000348:1), MADS box (cr.saCl001329:1), WRKY (cr.saCl000652:1), Zinc finger (cr.saCl000359) and ULT (cr.saCl000502:1). The expression pattern for all these genes was investigated in various tissue types and at different developmental stages using quantitative real time PCR. Our results demonstrated that a *ULT* transcription factor showed higher induction in stigma part of the flower and its expression increased till the day of anthesis and then subsequently decreased (Figure 2a and b). This expression pattern corroborated with the accumulation pattern of apocarotenoids suggesting involvement of this gene in regulating biosynthesis of these compounds. Among other genes studied, only *Myb* showed higher expression in stigma as compared to other flower parts, however, its expression at different developmental stages of stigma did not match with the pattern of apocarotenoid accumulation (Additional file 3).

**Figure 3:**
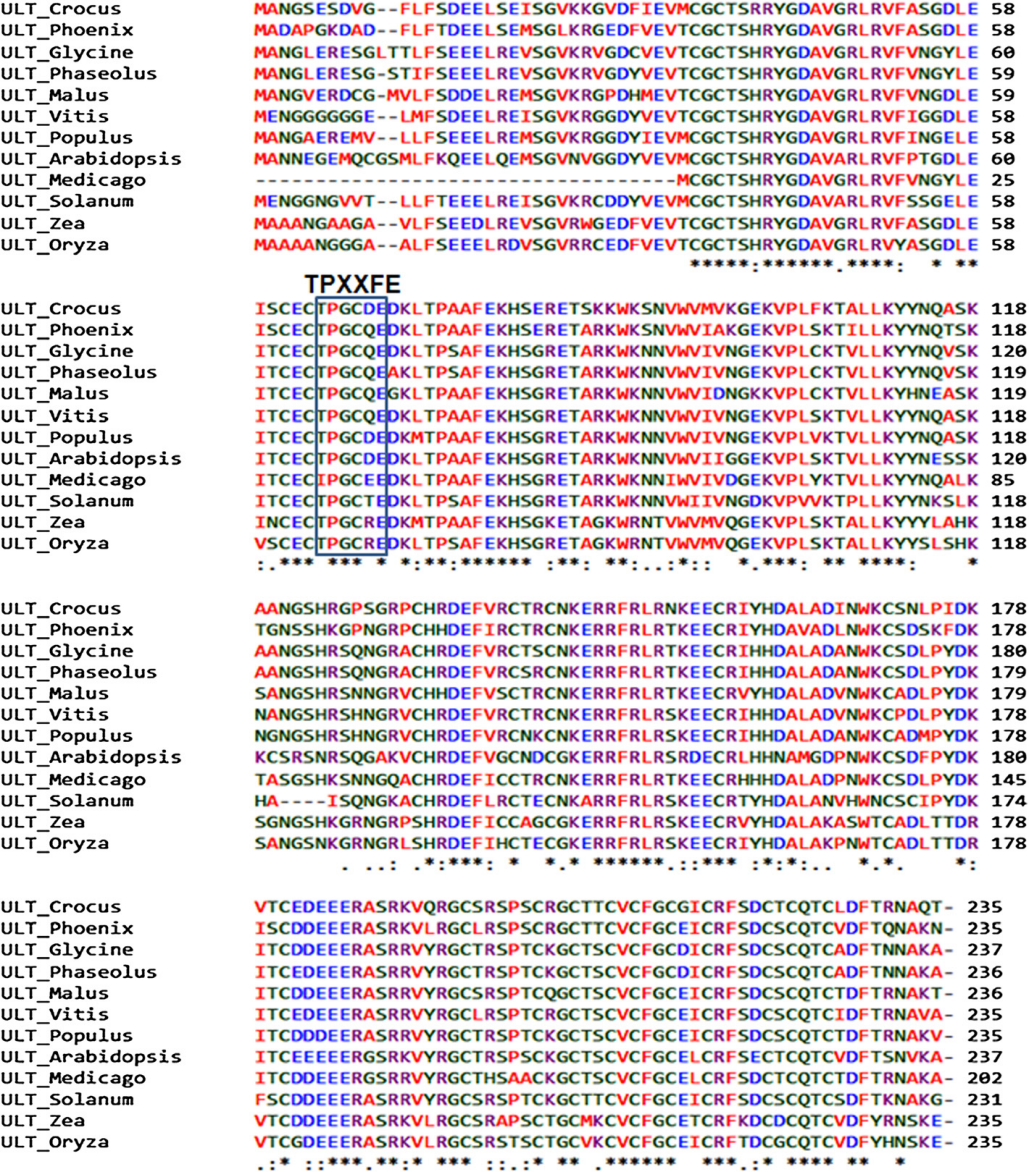
**Multiple sequence alignment of CsULT1.** The deduced amino acid sequence of CsULT1 is aligned with homologs from other plants.

**Figure 4:**
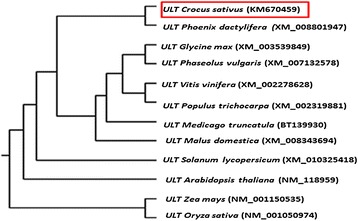
**Phylogenetic analysis of CsULT1.** A neighbor-joining phylogenetic tree of CsULT1 and selected ULT proteins from other plant species. The statistical reliability of individual nodes of the tree is assessed by bootstrap analyses with 1,000 replications.

Full length *ULT* was cloned by RT- PCR and 5′ RACE and was named as *CsULT1* [GenBank accession number: KM670459]. The gene contains 708 bp open reading frame coding for 235 amino acids long protein (Additional file 4) with a predicted molecular mass of 26.5kD and pI 8.32. Domain search revealed presence of conserved SAND domain in *CsULT1* which normally consists of evolutionarily conserved 80 to 100 amino acid long DNA binding motif [18]. The sequence alignment of ULTs from various organisms has revealed two conserved cores in SAND domains viz TPxxFE and KDWK. While TPxxFE is perfectly conserved among all ULT proteins in plants, KDWK shows variability at primary level however, the secondary structure is conserved [19]. Alignment of *CsULT1* with other plant ortholgs (Figure 3) showed high sequence homology with ULT from *Phoenix* (79.57%), *Vitis vinifera* (78%), *Populus* (76%) and *Medicago* (75%). Sequence alignment showed that ULT proteins show significant homology along the entire length of the protein except at the extreme N terminus. Moreover, TPxxFE motif was present in *CsULT1* and was conserved among all the proteins used for alignment. Phylogenetic analysis of selected sequences placed *CsULT1* close to its homolog from *Phoenix* (Figure 4).

### Subcellular localization of *CsULT1*

**Figure 5:**
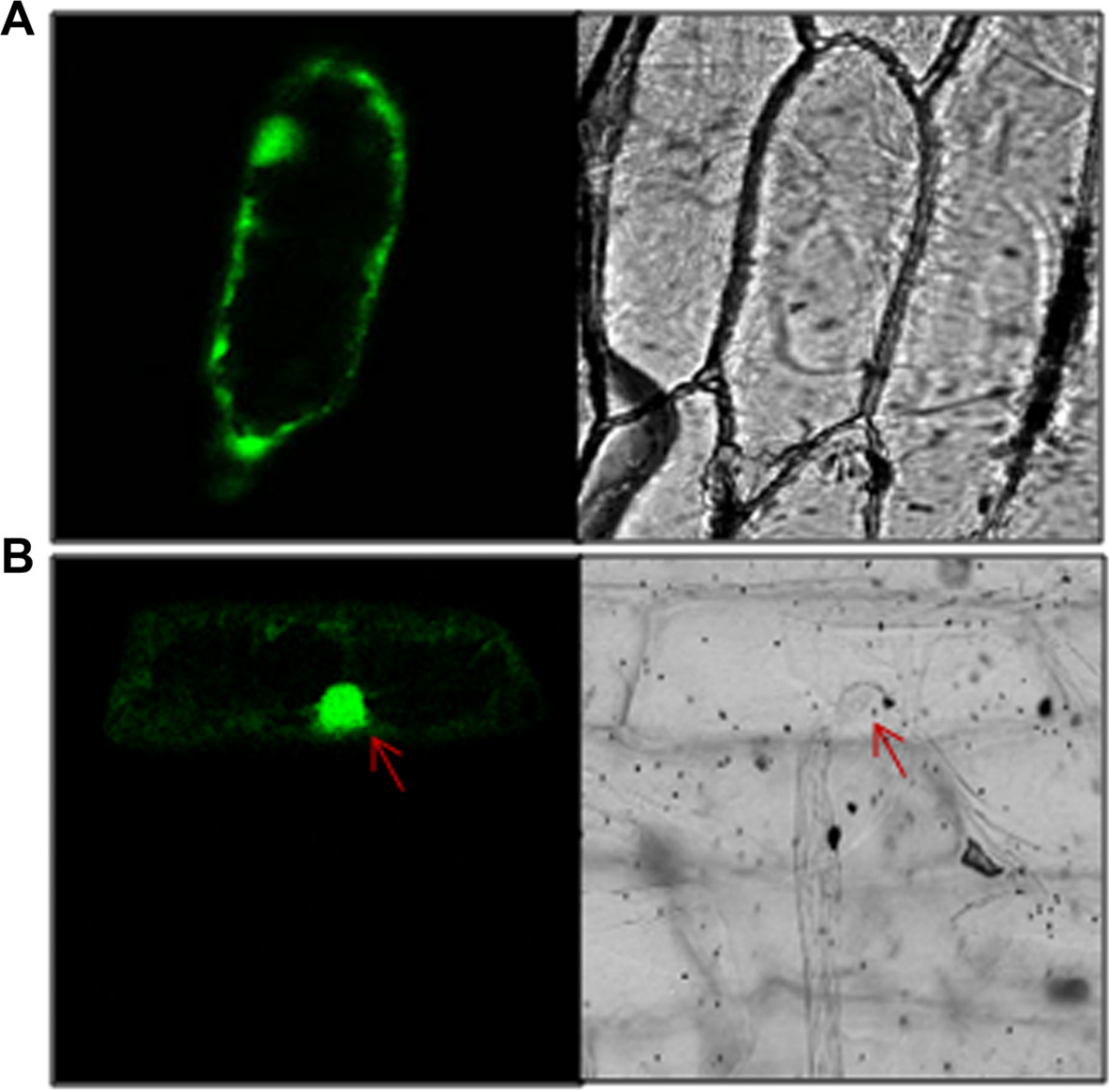
**Subcellular localization of** ***CsULT1*** **. (A)** GFP is accumulated throughout the cell **(B)** *CsULT1-GFP* is localized to the nucleus.

In order to have a preliminary understanding about the mechanism underlying the regulatory activity of *CsULT1*, its subcellular localization was investigated. Programs like Prosite and PSORT revealed absence of any sorting signal and predicted *CsULT1* to be localized in cytosol. For confirming the localization experimentally, *CsULT1* was cloned in frame with GFP reporter gene. The expression of the fusion gene construct *CsULT1*-GFP was driven by the 35S promoter of cauliflower mosaic virus (*CaMV-35S*). The fusion gene was introduced into onion (*Allium cepa)* epidermal cells by particle bombardment. While the control GFP accumulated throughout the cell, *CsULT1*-GFP was localized in the nucleus (Figure 5). This might be because of the fact that ULT proteins are small enough and can diffuse passively into the nucleus through the nuclear pores [15].

### Transactivation assay

**Figure 6:**
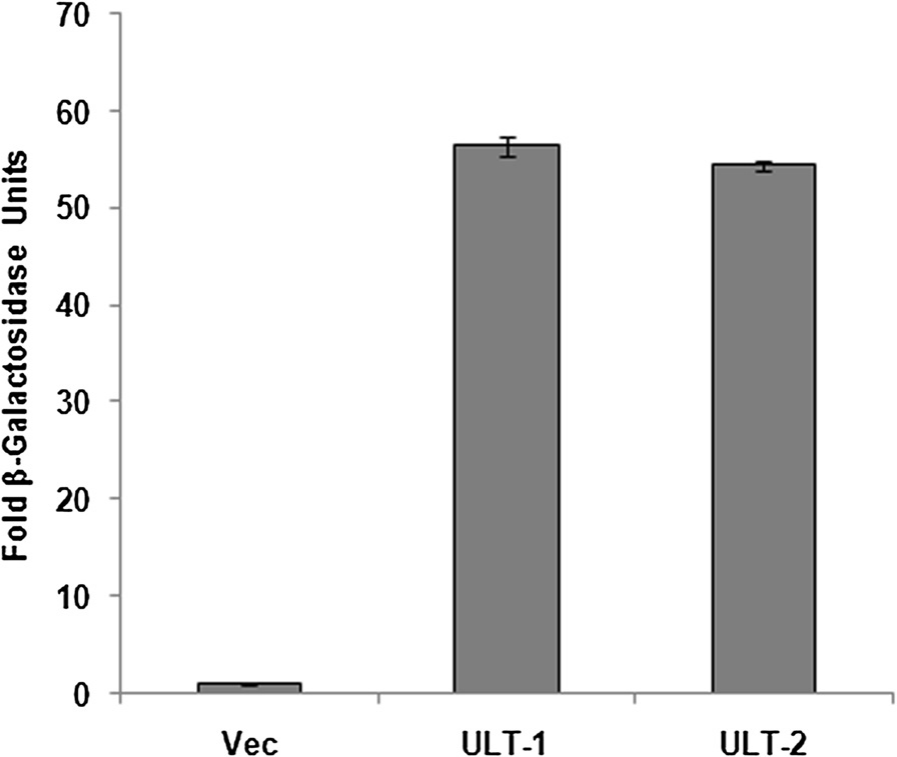
**Transactivation analysis of** ***CsULT1*** **by β-galactosidase assay.** Vec represents empty vector control and ULT-1 and ULT-2 represent two independent colonies used for the assay. Values are taken as average of three independent experiments of the transformants and presented as fold increase in activity.

To investigate the ability of *CsULT1* to activate transcription, a transient expression assay was performed using a GAL4-responsive reporter system in yeast cells. For this, the full-length coding region of *CsULT1* was fused to the GAL4 DNA-binding domain (BD) to generate pGBKT7-*CsULT1-BD* construct which was then transformed into yeast strain Y187. The transformants were assayed for their ability to activate transcription from the GAL4 upstream activation sequence. The transformed yeast cells harboring pGBKT7-*CsULT1-BD* construct grew well in SD medium lacking tryptophan and leucine, and showed β-galactosidase activity, whereas cells containing pGBKT7 (negative control) showed no β-galactosidase activity (Figure 6). This data confirmed transcriptional activity of *CsULT1*.

### Induction of *CsULT1* by phytohormones

**Figure 7:**
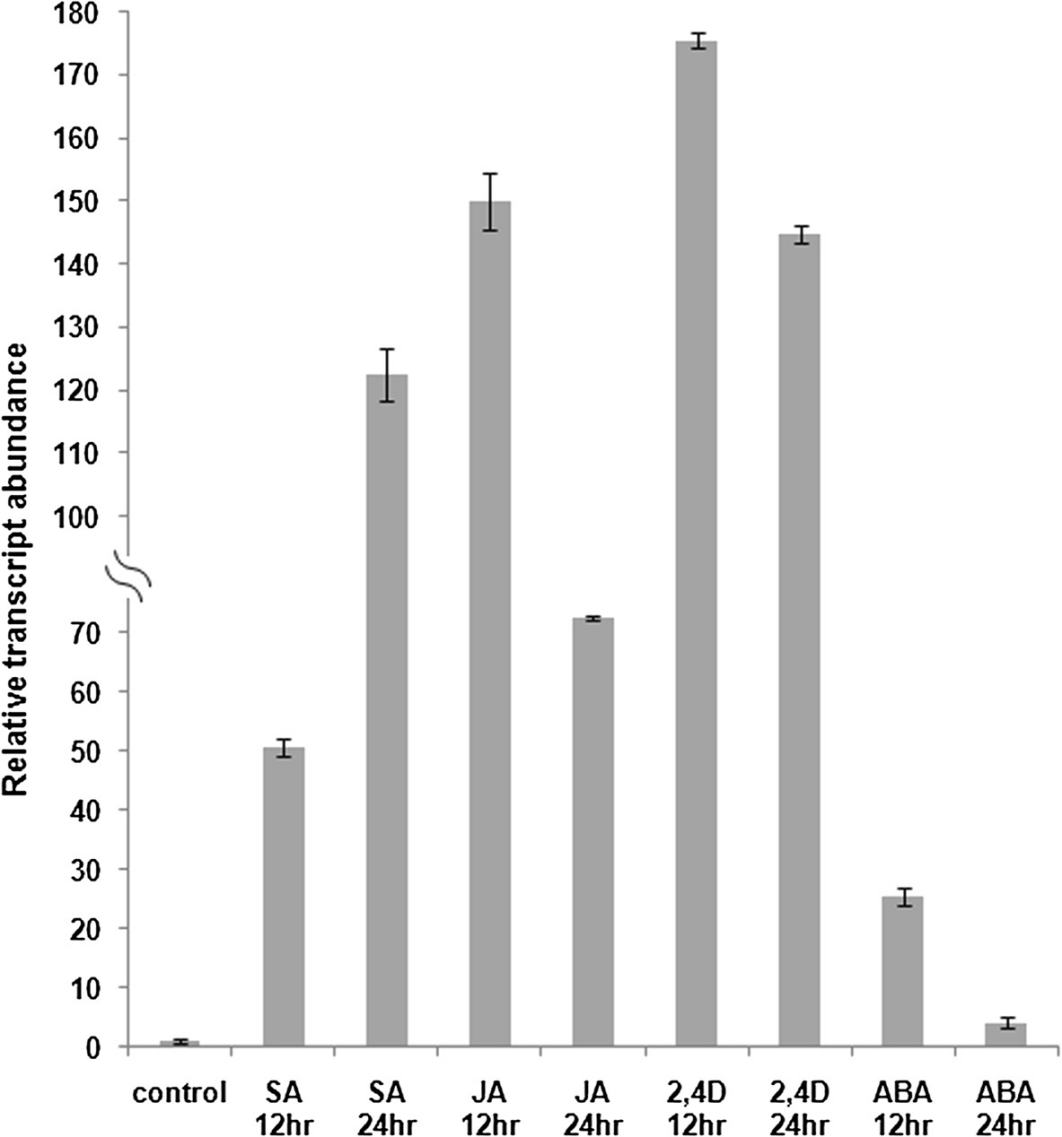
**qRT PCR showing relative transcript level of** ***CsULT1*** **in response to various hormones.** Transcript levels were normalized by actin transcript level. Error bars indicate SD of three replicates.

To investigate the effect of various phytohormones on expression of *CsULT1*, *Crocus* flowers were treated with salicylic acid (SA), methyljasmonate (JA), 2,4-D and abscisic acid (ABA) in a time course study. The expression of *CsULT1* was measured by qPCR using RNA isolated from treated tissue samples. Compared with the uninduced control, *CsULT1* expression increased in response to all these hormones used (Figure 7). In response to SA treatment, expression of *CsULT1* increased upto 122 fold (log 7 fold) at 24 hr post treatment while in response to JA, the expression enhanced approximately upto 150 fold (log 7 fold) at 12 h post treatment. *CsULT1* showed maximum change in expression in response to 2,4-D where it showed 175 fold (log 7.4) induction at 12 hr of treatment. However, there was not much significant change in expression in response to ABA.

### Transient over-expression of *CsULT1* in *C. sativus* calli increases MEP pathway gene expression

**Figure 8:**
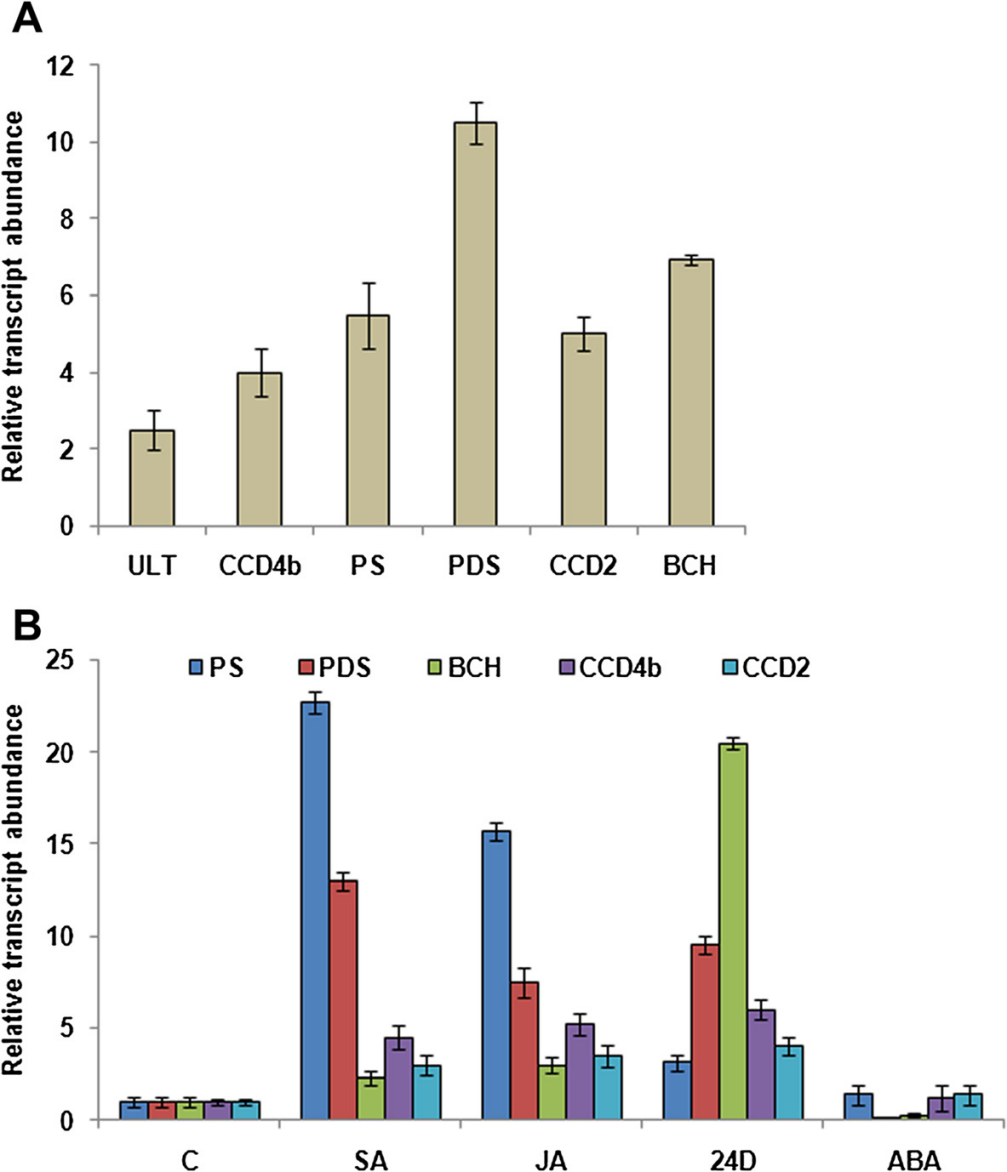
**Relative expression levels of selected carotenoid pathway genes (A) in** ***CsULT1*** **overexpressing calli (B) in response to various phytohormones.** The actin gene was used as an internal control. Each relative gene expression represents the average of three replicates with error bars representing SD.

For gaining an understanding on the role of *CsULT1* in *Crocus* apocarotenoid biosynthesis, the gene was transiently expressed in *Crocus* calli under the control of CaMV-35S promoter. The presence of transgene in transiently transformed calli was confirmed by genomic PCR. In the *CsULT1*-overexpressing calli, the *CsULT1* gene was expressed 2.5 fold higher than the empty vector control. Further, we checked expression of few of the MEP pathway genes and observed that *PSY*, *PDS, BCH, CsCCD4b* and *CsCCD*2 genes showed upregulation in *CsULT1* overexpressing calli (Figure 8a). *PSY* and *PDS* catalyze the initial rate limiting steps in carotenoid biosynthetic pathway. Further, *BCH* is involved in the formation of zeaxanthin from beta carotene [11] and this zeaxanthin acts as the substrate for the formation of *Crocus* apocarotenoids by *CsCCD2* enzyme [20]. *CsCCD4b* (another member of CCD gene family) is also involved in the formation of apocarotenoids from carotenoid substrates. Therefore, enhanced expression of the above mentioned genes may result in increased zeaxanthin pool which may subsequently be tailored to form apocarotenoids.

Various phytohormones were shown to induce expression of *CsULT1* which in turn induced expression of key pathway genes of carotenoid metabolism. Therefore we were keen to investigate change in expression of pathway genes in response to phytohormone application. It was observed that SA, JA and 2,4-D induced expression of carotenoid pathway genes (Figure 8b) therefore indicating their possible role in mediating the function of *CsULT1* in regulating carotenoid/apocarotenoid biosynthesis. Taken together, these results suggest that *CsULT1* has a role in regulating metabolic flux towards the biosynthesis of apocarotenoids in *Crocus*.

## Discussion

*Crocus* is known to accumulate large amounts of the apocarotenoids like crocetin (and its glycosylated forms, crocins), picrocrocins and saffranal in stigma part of the flowers. The proposed biosynthetic pathway is initiated through the symmetric cleavage of zeaxanthin at the 7,8/7,8 positions by a CCD2 enzyme [20]. The two cleavage products formed are 3-OH-β-cyclocitral and crocetin dialdehyde which are further dehydrogenated and glycosylated to yield picrocrocin and crocins respectively. Aim of our study was to identify transcription factors regulating synthesis of these *Crocus* apocarotenoids. For this, we used combined approach of transcript and metabolite profiling. Since crocetin (which is subsequently converted into crocin) and picrocrocin are products of same cleavage step and crocin is more stable, we investigated crocin levels in different parts of *Crocus* flower and in stigma collected at three different developmental stages (pre anthesis, anthesis and post anthesis). We could detect crocin only in stigma while in other parts it was below detection levels. Further, crocin content increased from pre anthesis stage to anthesis and later decreased post anthesis (Figure 1). In earlier reports also same trend has been described for crocin accumulation [12]. Next, expression profile of *CsULT1* was investigated which indicated that it follows the same trend (Figure 2) and corroborates with accumulation pattern of crocin suggesting its possible role in regulating the crocin biosynthetic pathway. The apocarotenoid accumulation varies with developmental stages and thus in order to fit into this narrow window of developmental changes, chromatin needs to be flexibly regulated so as to confer stable expression states that can be reset owing to changes in the progression of development. Since recently *ULT* has been shown to act in chromatin mediated pathways, its involvement in regulating plant secondary metabolic pathways would be a new dimension to its functional domain.

Domain analysis showed that *CsULT1* contains a SAND domain which represents conserved 80-residue amino acid sequence and is found in a number of nuclear proteins, many of which function in chromatin-dependent transcriptional control [13]. These include proteins linked to various human diseases, such as the Sp100 (Speckled protein 100 kDa), NUDR (Nuclear DEAF-1 related), GMEB (Glucocorticoid Modulatory Element Binding) proteins and AIRE-1 (Autoimmune regulator 1) proteins [18]. Many of these proteins have been shown to bind DNA, but no clear sequence or structural relationship to known DNA binding motifs has been established. Based on the conservation of positively charged residues, including a characteristic KDWK sequence motif, the SAND domain has been suggested to mediate the DNA binding of these proteins.

In animals SAND domain-containing proteins are found in nucleus or cytoplasm, or have dual localization being present in both nucleus and cytoplasm [21-23]. In confirmation with this data pertaining to animal proteins, plant ultrapetala proteins with SAND domains are also demonstrated to localize to both the nucleus and the cytosol [15]. However, our study showed that *CsULT1* is localized in nucleus (Figure 5). SAND domain containing proteins have been found to be transcriptionally active and are involved in regulation of gene expression [17]. We also investigated transcriptional activity of *CsULT1* and our results demonstrated that it activated beta galactosidase enzyme proving that it is transcriptionally active (Figure 6).

Plant developmental, metabolic and stress pathways have been shown to be influenced and controlled by various phytohormones. In order to gain an insight about effect of different phytohormones on *CsULT1* expression, we investigated effect of SA, JA, 2,4D and ABA on expression of *CsULT1*. Our results indicated that *CsULT1* is induced in response to all the hormones studied. However, effect of SA, JA and 2,4-D was much more profound than ABA (Figure 7). Jasmonates are known elicitors of plant secondary metabolism and trigger extensive transcriptional reprogramming which ultimately leads to activation of whole metabolic pathway [24]. Induction of *CsULT1* in response to JA might be part of this transcriptional activation scenario which as a final outcome leads to activation of carotenoid metabolic pathway. SA has been reported to induce expression of many carotenogenesis related genes [25]. Since *CsULT1* is a probable regulator of carotenogenesis, its induction in respone to SA is thus in confirmation with earlier reports. Auxin has been demonstrated to have a profound effect on stigma development [26]. Therefore enhanced expression of *CsULT1* in response to 2,4-D treatment might suggest a parallel role of auxin in regulating stigma development vis a vis apocarotenoid biosynthesis in *Crocus.* Although ABA treatment also enhanced expression of *CsULT1*, it was much less as compared to other hormones.

Several attempts have been made to establish *Crocus* transformation but no success has been achieved so far. Lack of transformation protocol is a limitation for functional characterization of genes in *Crocus*. The site of apocarotenoid biosynthesis is *Crocus* stigma, however, many of the pathway genes are expressed in callus also. Considering the limitation of transformation system, we transiently overexpressed *CsULT1* in *Crocus* calli by particle bombardment in order to confirm its role in regulating carotenoid/apocarotenoid pathway. The expression of *CsULT1* in transgenic calli was 2.5 fold as compared to vector control. This value is not good enough but since transformation in *Crocus* has not been established and callus is not the actual site of gene expression, the reported increase in expression can be considered as significant. Further, the expression analysis of a few carotenoid pathway genes was carried out and the results showed increase in expression level of *PSY* and *PDS* genes which catalyze initial rate limit steps of this pathway. This suggests role of *CsULT1* in regulating carotenoid biosynthesis in *Crocus*. Till so far there is only one report on regulation of *PSY* gene expression by phytochrome interacting factor (*PIF1*) which binds directly to *PSY* promoter and thereby regulates carotenoid accumulation during daily cycles of light and dark in mature plants [27]. Another member of AP2 gene family (*RAP2.2*) binds to of *PSY* promoter and is shown to modestly regulate the transcript levels of *PSY* and *PDS* in Arabidopsis [28]. Also *BCH* which is involved in conversion of beta carotene into zeaxanthin showed enhanced expression in transgenic calli (Figure 8a). In earlier reports *CsCCD4b* was considered responsible for cleaving zeaxanthin to produce apocarotenoids. However, later it was shown to cleave beta carotene at the 9,10 and/or the 9,10 positions, yielding beta-ionone. Recently a new isoform of CCDs (*CsCCD2*) was identified and isolated from *Crocus* and was shown to cleave zeaxanthin sequentially at 7,8 and 7,8 double bonds suggesting that *CsCCD2* catalyzes the first dedicated step in crocin biosynthesis [20]. We investigated change in expression of *CsCCD4b* as well as *CsCCD2* in transgenic calli and observed that their expression was enhanced around 4 and 5 fold respectively (Figure 8a). This suggests that apart from regulating synthesis of crocin and picrocrocin from zeaxanthin, *CsULT1* also plays role in regulating biosynthesis of other apocarotenoids including beta ionone. Thus *CsULT1* might regulate expression of more than one members of *CCD* gene family. Except for *PIF1* and *RAP2.2* no other transcription factors have been identified till so far which regulate expression of genes involved in carotenogenesis in plants. Therefore the present work will form a platform for enhancing our knowledege on regulation of this important pathway.

Carotenoids are involved in many biological functions including plastid biogenesis, photosynthesis, photomorphogenesis etc. Carotenoid metabolic pathway is also linked with many other pathways like ABA and GA biosynthesis. Therefore, carotenoid metabolism might be regulated at multifaceted levels in plants. Further, because of this close coordination of many pathways, content and composition of carotenoids is important. Thus biosynthesis of carotenoids and their turn-over to produce apocarotenoids needs to be tightly regulated in order to maintain their steady levels in plants. Hormones are known to play key roles in regulating various metabolic pathways. They also help in coordinating interplay between various pathways. Our results also demonstrated effect of phytohormones on *CsULT1* expression. In order to further our understanding on mechanism of regulation of carotenoid biosynthesis by *CsULT1*, we also investigated effect of various phytohormones on carotenoid pathway genes particularly those which showed enhanced expression in *CsULT1* overexpressing calli. Our results indicated that expression of all the genes studied was enhanced in response to SA, JA and 2, 4-D, however, there was no significant increase in expression of these genes in response to ABA (Figure 8b). Earlier also, SA and JA have been shown to affect expression of many carotenogenic pathway genes [29]. If considered individually, *PSY* was induced more in response to SA and JA while *PDS* expression increased more under the influence of SA and 2,4-D. *BCH* showed much higher response to 2,4-D than other hormones while *CCD4b* and *CCD2* showed more or less similar trend in expression in response to all the hormones except ABA. ABA did not cause any significant effect on expression of carotenoid pathway genes. Earlier, exogenous application of ABA was shown to repress transcript levels of many chloroplast genes [30]. It was also found to reduce chlorophyll levels and also endogenous ABA levels [31]. This might be the reason that we did not observe any significant increase in expression levels of carotenoid pathway genes in response to ABA. Thus in general, change in the expression of these genes followed the same trend as that of *CsULT1*. This suggests that these phytohormones might play a role in mediating effect of *CsULT1* on regulation of carotenoid metabolism. Further, different genes showed varied induction levels in response to different hormones which might be because of the fact that control of plant metabolic pathways is a complex phenomenon and network of cross-communicating hormone signaling pathways are involved so as to maintain overall metabolite homeostasis within the plant system.

Taken together, these results suggest that *CsULT1* plays role in diverting metabolic flux towards enhanced production of apocarotenoids. Recently *ULT* in *Arabidopsis* has been shown to be involved in gynoecium development and patterning [17]. In *Crocus*, apocarotenoid biosynthesis is congruent with stigma development. This indicates that *CsULT1* might have a parallel role in regulating *Crocus* gynoecium (stigma) development vis a vis apocarotenoid biosynthesis. However, this needs to be further verified by experimentation in *Crocus* itself or in other alternative plant system.

## Conclusions

In this study an ultrapetala transcription factor from *Crocus*, *CsULT1*, was identified, cloned and characterized. The *CsULT1* transcript was expressed more in stigma till flower anthesis. Application of phytohormones like 2,4D, JA and SA enhanced *CsULT1* expression. The gene is nuclear localized and is transcriptionally active. Moreover, ectopic expression of this gene altered expression of some important genes of carotenoid/apocarotenoid pathway confirming that *CsULT1* plays a regulatory role in *Crocus* apocarotenoid biosynthesis. Furthermore, the hormones which affected expression of *CsULT1* were also shown to enhance expression of pathway genes indicating their role in mediating regulatory role of *CsULT1*.

## Additional files

Additional file 1: Table S1. List of primer sequences. Additional file 2: Figure S1. Melt curve depicting single peak in qRT-PCR. Additional file 3: Figure S2. qRT-PCR analysis of different *Crocus* transcription factors. Additional file 4: Figure S3. Nucleotide and deduced amino acid sequence of *CsULT1.*

## Abbreviations

ULT: Ultrapetala
PSY: Phytoene synthase
PDS: Phytoene desaturase
CCD: Carotenoid cleavage dioxygenase
BCH: Beta cyclohydroxylase
MEP: Methylerythritol phosphate
