# Supplementary material for: Identification, cloning and characterization of an ultrapetala transcription factor CsULT1 from Crocus: a novel regulator of apocarotenoid biosynthesis
Source: BMC Plant Biol. 2015 Feb 1;15:25. doi: 10.1186/s12870-015-0423-7 (PMC4349709; doi:10.1186/s12870-015-0423-7)
Supplement: Additional file 1: Table S1. — List of primer sequences. [file 12870_2015_423_MOESM1_ESM.doc]

**Table S1. List of primer sequences**

| **S.No.** | **Name of Primer** | **Sequence of Primer** |
| --- | --- | --- |
| **1** | ULTRT-F | GCACTACCTGTGTCTGTTTTGGAT |
| **2** | ULTRT-R | GGTCTGGCAGGTGCAGTCA |
| **3** | Cs Myb-F | GGCACAGCAAAAGCATCAGTAC |
| **4** | CsMyb-R | GCAGAGCTACCAAATGGACATG |
| **5** | CsMAD-F | AGCCGAGCCAGCCAAAC |
| **6** | CsMAD-R | TCATGCAAGACGCAGATCATG |
| **7** | CsWR-F | CGTCGAAGGCAGACCAAAA |
| **8** | CsWR-R | ACTTGAACAGTACAACCGTTGATCA |
| **9** | Cs ZF-F | CCTCACAAGAGGAGCTTCGAA |
| **10** | Cs ZF-R | CCGGTGGCGAGTTGGTAA |
| **11** | ULT-F | GAGGTTTCGCCTCCGGAACA |
| **12** | ULT-R | TTAAGTCTGTGCATTCCTTG |
| **13** | ULT-5’ | GGAACATTTCCAGTTGATATCTGCCAAA |
| **14** | CsULT-F | ATGGCTAACGGGTCGGAGAG |
| **15** | CsULT-R | TTAAGTCTGTGCATTCCTTG |
| **16** | ULTCam-F | ATCATGCCATGGCTAACGGGTCGGAGAG |
| **17** | ULTCam-R1 | ATACGGACTAGTAGTCTGTGCATTCCTTG |
| **18** | ULTCam-R2 | ATACGGACTAGTTTAAGTCTGTGCATTCCTTG |
| **19** | ULTGBKT-F | GGAATTC CATATGGCTAACGGGTCGGAGAG |
| **20** | ULTGBKT-R | ATACC G GAAT TC TTAAGTCTGTGCATTCCTTG |
| **21** | PSY-F | GGCAGCATCGAACATGTCAT |
| **22** | PSY-R | GAGCCAATTGGAGGATCTT |
| **23** | PDS-F | CTGGTTGCGCGGAGTGA |
| **24** | PDS-R | TCCCTATTTAGGCAATGGTCA |
| **25** | BCH-F | TCGGAGCGGACCACGTA |
| **26** | BCH-R | TGGATGTGATGCCGAAGCT |
| **27** | CCD4b-F | CGCGATCACCGAACACTATG |
| **28** | CCD4b-R | CGCGATCATCACCGAACACTAT |
| **29** | CCD2-F | AAGTTTGCTCCCGTAGCCGGATA |
| **30** | CCD2-R | CCTTAGATCTCCAATCTTCAT |
| **31** | Actin-F | GCATAAAGAGACAGGACGGC |
| **32** | Actin-R | TCGTGTGGCTCCCGAGGAGC |
| **33** | GFP-R | CTAGCATTCGCCATTCAGGCTGCGC |
