# Supplementary figures and images for: Identification, cloning and characterization of an ultrapetala transcription factor CsULT1 from Crocus: a novel regulator of apocarotenoid biosynthesis
Source: BMC Plant Biol. 2015 Feb 1;15:25. doi: 10.1186/s12870-015-0423-7 (PMC4349709; doi:10.1186/s12870-015-0423-7)

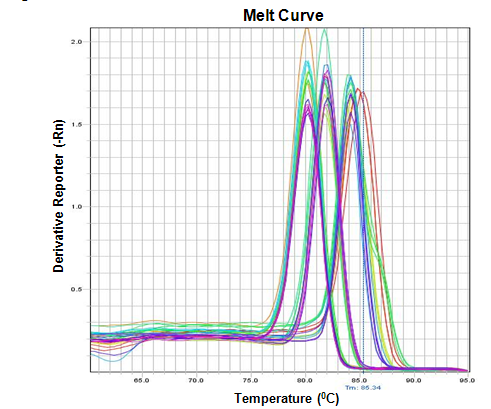

Supplement: Additional file 2: Figure S1. — Melt curve depicting single peak in qRT-PCR. [file 12870_2015_423_MOESM2_ESM.tiff]

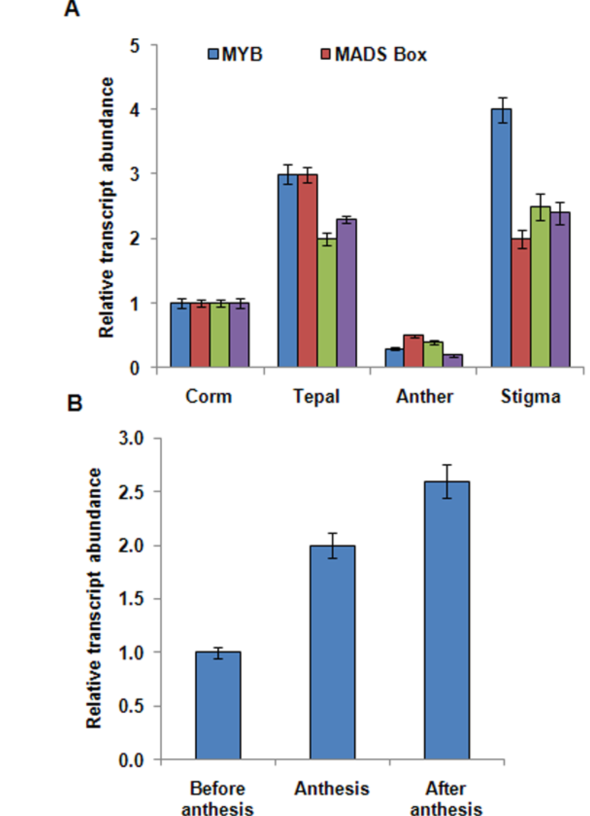

Supplement: Additional file 3: Figure S2. — qRT-PCR analysis of different Crocus transcription factors. [file 12870_2015_423_MOESM3_ESM.tiff]

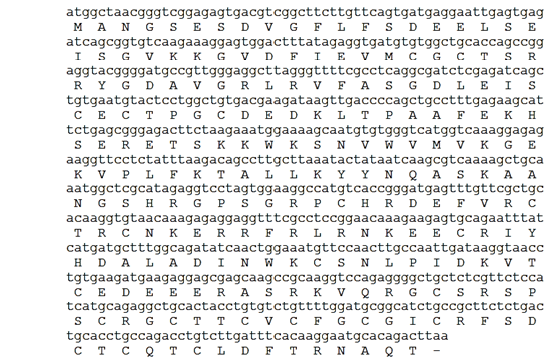

Supplement: Additional file 4: Figure S3. — Nucleotide and deduced amino acid sequence of CsULT1. [file 12870_2015_423_MOESM4_ESM.tiff]
